# Benzo[1,2-*b*:4,5-*b*′]dithio­phene-4,8-dione

**DOI:** 10.1107/S1600536812015826

**Published:** 2012-04-18

**Authors:** Amanda L. Ramirez, Benny C. Chan, Daniel T. de Lill

**Affiliations:** a Department of Chemistry & Biochemistry, Florida Atlantic University, 777 Glades Road, Boca Raton, FL 33431, USA; b Department of Chemistry, The College of New Jersey, Ewing, NJ 08628, USA

## Abstract

The title mol­ecule, C_10_H_4_O_2_S_2_, is situated on a crystallographic center of inversion. In the crystal, weak hydrogen bonding contributes to the packing of the mol­ecules.

## Related literature

This dione was synthesized according to modified literature procedures, see: Beimlung & Kossmehl (1986 ▶); Slocum & Gierer (1976 ▶). It is a precursor to many different semiconducting polymeric compounds and the structure is important in that it appears as crystalline products in poorly purified materials, see: Hundt *et al.* (2009 ▶); Subramaniyan *et al.* (2011 ▶); Yamamoto *et al.* (2011 ▶). For weak inter­molecular inter­actions, see: Janiak (2000 ▶); Sinnokrot *et al.* (2002 ▶).
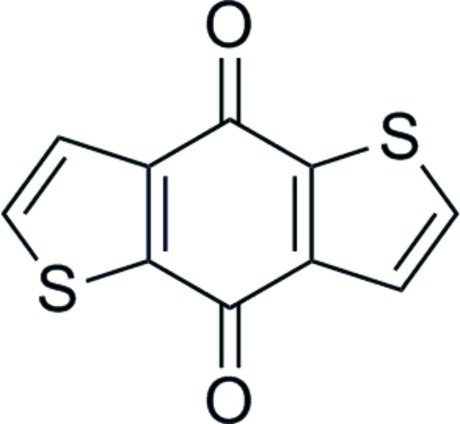


## Experimental

### 

#### Crystal data

C_10_H_4_O_2_S_2_
*M* *_r_* = 220.25
Monoclinic, 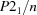

*a* = 5.6402 (5) Å
*b* = 5.7745 (5) Å
*c* = 13.6223 (12) Å
β = 97.371 (1)°
*V* = 440.00 (7) Å^3^
*Z* = 2
Mo *K*α radiation
μ = 0.57 mm^−1^
*T* = 296 K
0.08 × 0.06 × 0.04 mm

#### Data collection

Bruker APEXII diffractometer
Absorption correction: multi-scan (*SADABS*; Sheldrick, 2001 ▶) *T*_min_ = 0.955, *T*_max_ = 0.978
4888 measured reflections
1062 independent reflections
815 reflections with *I* > 2σ(*I*)
*R*_int_ = 0.072

#### Refinement

*R*[*F* ^2^ > 2σ(*F* ^2^)] = 0.068
*wR*(*F* ^2^) = 0.238
*S* = 1.09
1062 reflections
64 parameters
H-atom parameters constrained
Δρ_max_ = 0.78 e Å^−3^
Δρ_min_ = −0.57 e Å^−3^

### 

Data collection: *APEX2* (Bruker, 2010 ▶); cell refinement: *SAINT* (Bruker, 2009 ▶); data reduction: *SAINT*; program(s) used to solve structure: *SHELXS97* (Sheldrick, 2008 ▶); program(s) used to refine structure: *SHELXL97* (Sheldrick, 2008 ▶); molecular graphics: *CrystalMaker* (Palmer, 2009 ▶); software used to prepare material for publication: *SHELXTL* (Sheldrick, 2008 ▶).

## Supplementary Material

Crystal structure: contains datablock(s) I, global. DOI: 10.1107/S1600536812015826/rn2094sup1.cif

Structure factors: contains datablock(s) I. DOI: 10.1107/S1600536812015826/rn2094Isup2.hkl

Supplementary material file. DOI: 10.1107/S1600536812015826/rn2094Isup3.cml

Additional supplementary materials:  crystallographic information; 3D view; checkCIF report

## Figures and Tables

**Table 1:** Hydrogen-bond geometry (Å, °)

*D*—H⋯*A*	*D*—H	H⋯*A*	*D*⋯*A*	*D*—H⋯*A*
C5—H5⋯O1^i^	0.93	2.44	3.319 (4)	158

Symmetry code: (i) 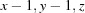.

**Fig. 1.**
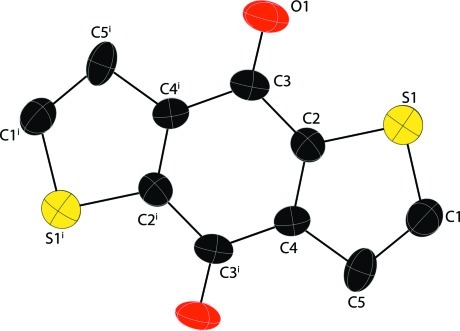
: Thermal ellipsoid plot (50% probablility) of title compound. Hydrogen atoms are omitted. Symmetry operator: i=-x + 2, -y + 1, -z + 2.

**Fig. 2.**
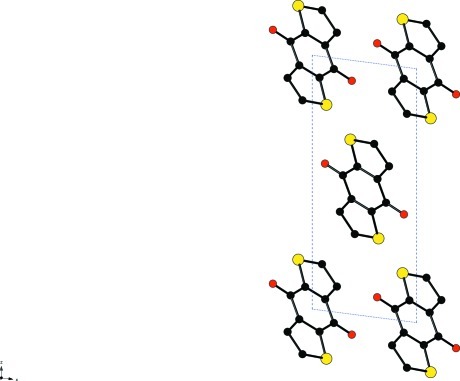
: Packing diagram of title compound down [010]. Hydrogen atoms were removed for clarity.

## supplementary crystallographic information

### Comment

Benzodithiophene-4,8-dione (BDTD) is a common precursor for the construction of semiconducting organic monomers. Given our interest in this field, we have isolated single crystals of this compound for structural determination. This structure is important in that it appears as crystalline products in poorly purified materials.

A thermal ellipsoid plot (Fig. 1) displays the molecular structure of the title compound.

Figure 2 shows a packing diagram of the crystal structure. Weak intermolecular interactions attribute to the packing of this compound, see: Sinnokrot *et al.* (2002); Janiak (2000). The closest CH···centroid distance is C1—H1···*Cg*1 at 3.715 (4) Å between a hydrogen atom and the center of a neighboring thiophene ring. No classic hydrogen bonds were found, but a weak hydrogen bond from C5—H5···O1 is present at 3.319 (4) Å D—A distance.

### Experimental

The title compound was prepared according to a modified literature procedure (Beimlung & Kossmehl, 1986). In a typical reaction, 2 g of 3-theonic acid was reacted with excess thionyl chloride (50 ml) at reflux temperature overnight to produce the resulting acid chloride. Upon removal of the thionyl chloride, the acid chloride was dissolved in toluene (minimum amount). The acid chloride was then added to excess diethylamine (approximately 42 ml) to produce the thiophene amide. The product was isolated in diethyl ether, concentrated, and then re-dissolved in ether (30 ml). The amide was then cyclized with excess *n*-butyl lithium (1.6 *M* in hexane, approximately 7 ml) added dropwise and allowed to stir overnight. The reaction was quenched with water, filtered, and recrystallized from glacial acetic acid in a 31–45% yield depending on reaction.

Upon isolation of the final compound, crystals suitable for X-ray diffraction were obtained from slow evaporation of approximately 20 mg of product in approximately 5 ml of chloroform. Clear, yellow block crystals formed overnight.

### Refinement

Crystallography. Hydrogen atom positions were placed in calculated positions and allowed to ride on the coordinates of the parent atom [C—H distances at 0.93 Å and *U*_iso_(H)=1.2*U*_iso_(C)].

### Crystal data

**Table:**

C_10_H_4_O_2_S_2_	*F*(000) = 224
*M**_r_* = 220.25	*D*_x_ = 1.662 Mg m^−^^3^
Monoclinic, *P*2_1_/*n*	Mo *K*α radiation, λ = 0.71073 Å
*a* = 5.6402 (5) Å	Cell parameters from 2545 reflections
*b* = 5.7745 (5) Å	θ = 6.0–55.2°
*c* = 13.6223 (12) Å	µ = 0.57 mm^−^^1^
β = 97.371 (1)°	*T* = 296 K
*V* = 440.00 (7) Å^3^	Prismatic block, clear yellow
*Z* = 2	0.08 × 0.06 × 0.04 mm

### Data collection

**Table:**

Bruker APEXII diffractometer	1062 independent reflections
Radiation source: fine-focus sealed tube	815 reflections with *I* > 2σ(*I*)
Graphite monochromator	*R*_int_ = 0.072
Fixed Chi scans	θ_max_ = 28.6°, θ_min_ = 3.0°
Absorption correction: multi-scan (*SADABS*; Sheldrick, 2001)	*h* = −7→7
*T*_min_ = 0.955, *T*_max_ = 0.978	*k* = −7→7
4888 measured reflections	*l* = −18→17

### Refinement

**Table:**

Refinement on *F*^2^	Primary atom site location: structure-invariant direct methods
Least-squares matrix: full	Secondary atom site location: difference Fourier map
*R*[*F*^2^ > 2σ(*F*^2^)] = 0.068	Hydrogen site location: inferred from neighbouring sites
*wR*(*F*^2^) = 0.238	H-atom parameters constrained
*S* = 1.09	*w* = 1/[σ^2^(*F*_o_^2^) + (0.1499*P*)^2^ + 0.2282*P*] where *P* = (*F*_o_^2^ + 2*F*_c_^2^)/3
1062 reflections	(Δ/σ)_max_ < 0.001
64 parameters	Δρ_max_ = 0.78 e Å^−^^3^
0 restraints	Δρ_min_ = −0.57 e Å^−^^3^

### Special details

**Table:**

Geometry. All e.s.d.'s (except the e.s.d. in the dihedral angle between two l.s. planes) are estimated using the full covariance matrix. The cell e.s.d.'s are taken into account individually in the estimation of e.s.d.'s in distances, angles and torsion angles; correlations between e.s.d.'s in cell parameters are only used when they are defined by crystal symmetry. An approximate (isotropic) treatment of cell e.s.d.'s is used for estimating e.s.d.'s involving l.s. planes.
Refinement. Refinement of *F*^2^ against ALL reflections. The weighted *R*-factor *wR* and goodness of fit *S* are based on *F*^2^, conventional *R*-factors *R* are based on *F*, with *F* set to zero for negative *F*^2^. The threshold expression of *F*^2^ > σ(*F*^2^) is used only for calculating *R*-factors(gt) *etc*. and is not relevant to the choice of reflections for refinement. *R*-factors based on *F*^2^ are statistically about twice as large as those based on *F*, and *R*- factors based on ALL data will be even larger.

### Fractional atomic coordinates and isotropic or equivalent isotropic displacement parameters (Å^2^)

**Table:**

	*x*	*y*	*z*	*U*_iso_*/*U*_eq_	
S1	0.13427 (18)	0.25931 (18)	0.81226 (8)	0.0558 (5)	
O1	0.3782 (5)	0.6780 (5)	0.9199 (2)	0.0540 (8)	
C1	−0.0936 (7)	0.0712 (6)	0.8176 (3)	0.0517 (9)	
H1	−0.1261	−0.0467	0.7714	0.062*	
C2	0.0689 (6)	0.3977 (5)	0.9156 (2)	0.0379 (7)	
C3	0.2074 (6)	0.5981 (5)	0.9569 (2)	0.0390 (8)	
C4	−0.1243 (5)	0.3053 (5)	0.9539 (2)	0.0366 (7)	
C5	−0.2363 (6)	0.1035 (5)	0.8983 (2)	0.0421 (8)	
H5	−0.3675	0.0174	0.9120	0.050*	

### Atomic displacement parameters (Å^2^)

**Table:**

	*U*^11^	*U*^22^	*U*^33^	*U*^12^	*U*^13^	*U*^23^
S1	0.0531 (8)	0.0612 (8)	0.0549 (8)	−0.0008 (4)	0.0137 (5)	−0.0091 (4)
O1	0.0442 (14)	0.0666 (17)	0.0540 (15)	−0.0147 (12)	0.0175 (11)	0.0106 (13)
C1	0.0493 (19)	0.0468 (19)	0.058 (2)	−0.0021 (15)	0.0037 (16)	−0.0143 (16)
C2	0.0371 (15)	0.0388 (16)	0.0384 (15)	0.0010 (12)	0.0079 (12)	0.0028 (12)
C3	0.0345 (15)	0.0416 (16)	0.0413 (16)	−0.0041 (13)	0.0066 (12)	0.0096 (13)
C4	0.0339 (16)	0.0348 (15)	0.0406 (17)	−0.0034 (12)	0.0037 (13)	0.0043 (12)
C5	0.0534 (19)	0.0295 (14)	0.0394 (16)	0.0089 (13)	−0.0091 (14)	−0.0053 (12)

### Geometric parameters (Å, º)

**Table:**

S1—C1	1.691 (4)	C2—C3	1.467 (5)
S1—C2	1.700 (3)	C3—C4^i^	1.468 (5)
O1—C3	1.232 (4)	C4—C3^i^	1.468 (5)
C1—C5	1.455 (5)	C4—C5	1.486 (4)
C1—H1	0.9300	C5—H5	0.9300
C2—C4	1.374 (4)		
			
C1—S1—C2	91.10 (17)	O1—C3—C4^i^	123.0 (3)
C5—C1—S1	116.6 (3)	C2—C3—C4^i^	114.0 (3)
C5—C1—H1	121.7	C2—C4—C3^i^	121.3 (3)
S1—C1—H1	121.7	C2—C4—C5	114.7 (3)
C4—C2—C3	124.7 (3)	C3^i^—C4—C5	124.0 (3)
C4—C2—S1	113.5 (3)	C1—C5—C4	104.2 (3)
C3—C2—S1	121.8 (2)	C1—C5—H5	127.9
O1—C3—C2	123.0 (3)	C4—C5—H5	127.9

Symmetry code: (i) −*x*, −*y*+1, −*z*+2.

### Hydrogen-bond geometry (Å, º)

**Table:**

*D*—H···*A*	*D*—H	H···*A*	*D*···*A*	*D*—H···*A*
C5—H5···O1^ii^	0.93	2.44	3.319 (4)	158

Symmetry code: (ii) *x*−1, *y*−1, *z*.
